# Non-random retention of protein-coding overlapping genes in Metazoa

**DOI:** 10.1186/1471-2164-9-174

**Published:** 2008-04-16

**Authors:** Giulia Soldà, Mikita Suyama, Paride Pelucchi, Silvia Boi, Alessandro Guffanti, Ermanno Rizzi, Peer Bork, Maria Luisa Tenchini, Francesca D Ciccarelli

**Affiliations:** 1 Department of Biology and Genetics for Medical Sciences, University of Milan, 20133 Milan, Italy; 2 Center for Genomic Medicine, Kyoto University Graduate School of Medicine, Konoe-cho, Yoshida, Sakyo-ku, 606-8501 Kyoto, Japan; 3 Institute of Biomedical Technologies, National Research Council, Via Fantoli 16/15, 20138 Milan, Italy; 4 European Molecular Biology Laboratory, Meyerhofstr.1, 69012 Heidelberg, Germany; 5 Department of Experimental Oncology, European Institute of Oncology, Via Ripamonti 435, 20141 Milan, Italy; 6 FIRC Institute of Molecular Oncology Foundation, Via Adamello 16, 20139 Milan, Italy

## Abstract

**Background:**

Although the overlap of transcriptional units occurs frequently in eukaryotic genomes, its evolutionary and biological significance remains largely unclear. Here we report a comparative analysis of overlaps between genes coding for well-annotated proteins in five metazoan genomes (human, mouse, zebrafish, fruit fly and worm).

**Results:**

For all analyzed species the observed number of overlapping genes is always lower than expected assuming functional neutrality, suggesting that gene overlap is negatively selected. The comparison to the random distribution also shows that retained overlaps do not exhibit random features: antiparallel overlaps are significantly enriched, while overlaps lying on the same strand and those involving coding sequences are highly underrepresented. We confirm that overlap is mostly species-specific and provide evidence that it frequently originates through the acquisition of terminal, non-coding exons. Finally, we show that overlapping genes tend to be significantly co-expressed in a breast cancer cDNA library obtained by 454 deep sequencing, and that different overlap types display different patterns of reciprocal expression.

**Conclusion:**

Our data suggest that overlap between protein-coding genes is selected against in Metazoa. However, when retained it may be used as a species-specific mechanism for the reciprocal regulation of neighboring genes. The tendency of overlaps to involve non-coding regions of the genes leads to the speculation that the advantages achieved by an overlapping arrangement may be optimized by evolving regulatory non-coding transcripts.

## Background

The occurrence of overlapping genes in higher eukaryotes has long been considered a rare event [1,2], but the completion of genome sequencing efforts and whole-transcriptome analyses have instead revealed that mammalian genomes harbor a high number of overlapping transcriptional units [3-8]. The majority of detected overlaps occurs between genes transcribed from opposite strands of the same genomic locus and often involves non-coding RNAs [6,9-14]. These antisense transcripts participate in a number of cellular processes, such as genomic imprinting, X chromosome inactivation, alternative splicing, gene silencing and methylation, RNA editing and translation [15-20]. Comparatively, very little is known about overlapping genes lying on the same DNA strand, apart from a few cases reported in the literature [21-24]. Overlap is estimated to involve around 10% of protein-coding genes [13,25], raising to 20%–60% when non-coding RNAs are included [6,8-10,12,14,26,27]. Despite their abundance, the origin and evolution of overlapping genes in eukaryotes remain unclear, and different comparative studies have often led to discordant results [6,12-14,25]. The inclusion of non-coding RNAs and poorly annotated transcripts in these analyses, together with protein-coding genes, may have contributed to the conflicting results, as protein-coding genes and functional non-coding RNAs evolve differently [28]. In order to investigate the evolution of gene overlap in Metazoa we decided to use a dataset restricted to well-annotated protein-coding genes. We retrieved overlapping protein-coding genes in 5 representative species (*Homo sapiens*, *Mus musculus*, *Danio rerio*, *Drosophila melanogaster *and *Caenorhabditis elegans*), and compared the observed cases with a random distribution expected in case of functional neutrality. We identified features and conservation of protein-coding overlapping genes, and inferred possible mechanisms responsible for overlap formation. Finally, to evaluate the possible relationship between overlap and gene expression, we analyzed the expression of our set of overlapping genes in a human breast cancer cDNA library derived by 454 deep sequencing.

## Results and Discussion

### Non-random retention of protein-coding overlapping genes in Metazoa

**Figure 1:**
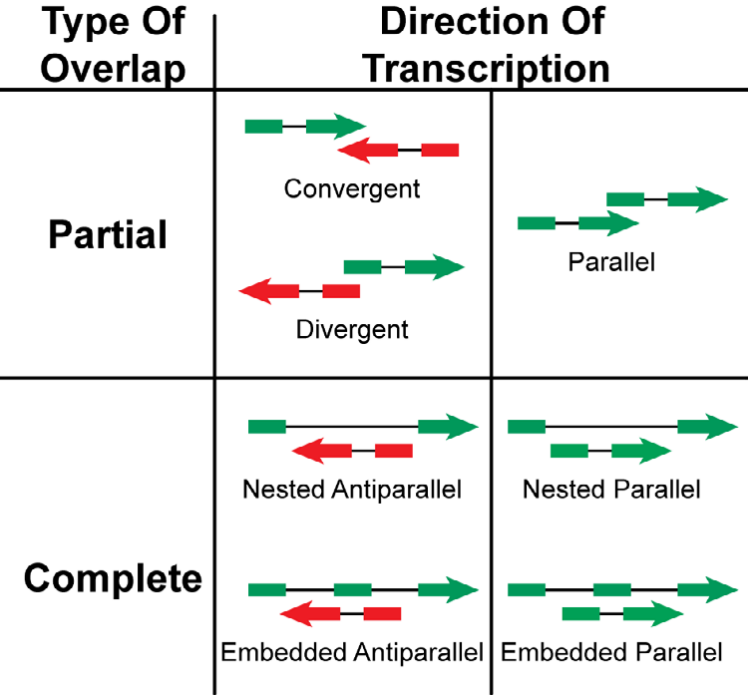
**Classes of overlapping genes**. OGC classification was based on the overlap extent (complete or partial) and on the reciprocal direction of transcription of the involved genes (same or opposite strand). Convergent overlaps involve the 3' termini of both genes, while divergent overlaps involve the 5' ends (UTR and/or CDS). Complete overlap occurs when the entire sequence of one gene is contained within another gene. In nested OGCs one gene lies completely within an intron of the other, while embedded genes can share more than one intron or exon.

The sequences of known protein-coding genes for five fully sequenced metazoan genomes (*H. sapiens, M. musculus, D. rerio, D. melanogaster, C. elegans*) were retrieved from several sources (RefSeq v.10, UCSC mm7 assembly, WormBase WS140, Flybase r4.2, Riken Fantom 3.0). From each dataset, we filtered splice variants and removed non-coding transcripts, pseudogenes and purely computational gene predictions, and mapped each cDNA on the corresponding genome to extract the Overlapping Gene Clusters (OGCs). OGCs were detected when there was partial or total overlap between the genomic coordinates of two or more genes. Gene boundaries were defined as the start and the end of the longest transcript (the complete list and features of OGCs are provided in Additional files 1 and 2). Our selection criteria allowed the detection of OGCs laying both on the same (parallel) and on opposite (antiparallel) DNA strand (Figure 1). Although we started from restrictive datasets, our estimates of overlapping protein-coding genes (Table 1) were consistent with previous analyses in human, mouse and Drosophila [13,27,29-31]. According to our results, overlap involves 4–8% of protein-coding genes, with the exception of Drosophila, where the percentage of OGCs is higher (26.2%, Table 1).

**Table 1:** Overlapping genes in five Metazoa.

**Species**	**Total Genes**	**Unique Genes**	**Observed OGCs**	**Expected OGCs (SD)**	**Observed OG Pairs**	**Expected OG Pairs (SD)**	**Observed OGs**	**Observed OGs (%)**	**Expected OGs (SD)**	**Expected OGs (%)**
*Hs*	23073	17794	663	2374.1 (27.7)	749	4954.0 (65.2)	1409	7.9	6630.8 (47.2)	37.3
*Mm*	17970	17040	656	2112.9 (27.9)	662	4293.7 (71.3)	1400	8.2	5873.7 (68.8)	34.5
*Dr*	6672	6506	108	396.9 (14.7)	155	524.2 (19.5)	262	4.0	899.1 (30.6)	13.8
*Dm*	18768	13416	1505	2172.3 (8.1)	2022	7483.1 (44.0)	3514	26.2	7876.0 (34.9)	58.7
*Ce*	21124	19359	404	3615.6 (32.2)	494	8653.1 (80.7)	898	4.6	10442.9 (54.3)	53.9

Unique Genes refer to the actual number of sequences used in the analysis, after filtering for splice variants. For each species, the counts of overlapping genes (OGs), overlapping gene pairs (OG pairs), and overlapping gene clusters (OGCs) coming from both real data and random simulations are shown. In the latter case the average number over ten simulations is reported together with the standard deviation (SD). Abbreviations: *Hs*, Homo sapiens;*Mm*, Mus musculus;*Dr*, Danio rerio;*Dm*, Drosophila melanogaster; *Ce*, Caenorabditis elegans.

We compared the observed data on overlapping genes to a null model that simulates the distribution of expected events in case of neutrality. For each species, we re-assigned random positions to the individual genes within each chromosome and counted the resulting number of overlaps.

In all species the overall number of observed OGCs was significantly lower than randomly expected (Table 1), suggesting selection against the retention of overlap as a general mechanism of gene arrangement. There are at least two reasons possibly explaining the counter selection of gene overlap in Metazoa. First, each mutation occurring within the overlapping regions would affect two or more sequences at the same time, and would likely reduce the ability of the involved genes to become optimally adapted [32]. Second, overlap can result in transcriptional [33,34] or translational [35] interference between overlapping reading frames. Both these reasons help to explain why OGCs formed by several genes, as well as those involving coding sequences, are particularly selected against (see below).

**Figure 2:**
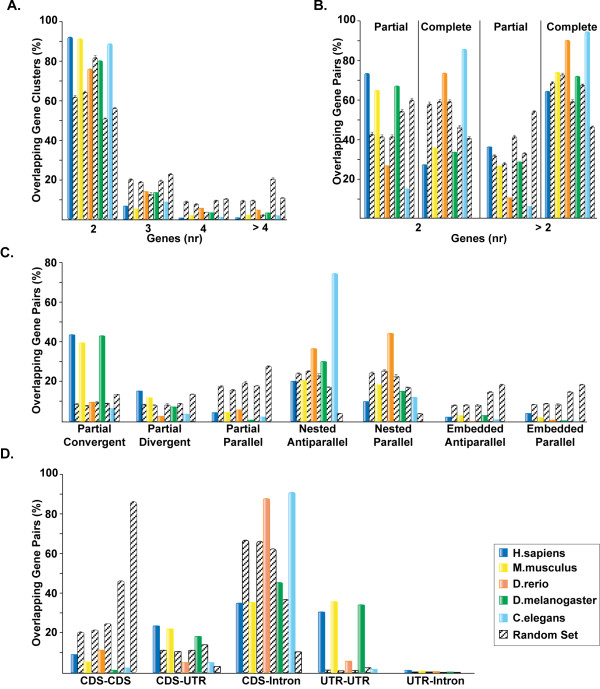
**Comparative analysis of OGCs in Metazoa**. For all species, the bar corresponding to each analyzed feature of the observed overlapping gene sets is followed by the bar corresponding to the random expectation. Since the simulations were repeated ten times, the corresponding standard deviation is associated to the random bars. **A**. OGC composition. OGCs were analyzed on the basis of the number of genes composing each cluster. The OGCs with more than 4 components are 5 in human, 11 in mouse, 5 in zebra fish, 48 in fly and 7 in worm. **B**. Type of overlap. Occurrence of partial and complete overlaps in both 2-component and multicomponent OGCs. **C**. Gene reciprocal arrangement. Distribution of OGCs according to the overlap type (refer to Figure 1). **D**. Features of the overlapping regions. The plot reports the number of overlaps involving coding sequence for one (CDS/UTR or CDS/intron overlaps) or both genes and the number of overlap involving only noncoding sequence (UTR/UTR and UTR/intron).

Although overlap of protein-coding genes is generally counterselected, some classes of overlap are preferentially retained. Comparison to random expectation showed that observed OGCs display a non-random distribution in terms of their abundance, reciprocal orientation, and overlap pattern (Table 1 and Figure 2).

While the number of random OGCs varied according to the different gene density of the analyzed species (Table 1 and Additional file 2), this tendency was not maintained in the observed data. Observed OGCs in human and mouse were around 4–5 times less than expected, while they were ~2 times less in fly and ~12 times less in worm. In agreement with our observation, a remarkable abundance of antisense transcripts in fly and a paucity in worm have been recently reported [12,14]. The different rates of overlapping genes in fly and worm could be due to species-specific features. The higher proportion of overlapping genes in fly might be partly explained by the high gene density and the extended UTR length (Additional File 1). The low number of OGCs in worm may be instead a consequence of the presence of operons, which involve at least 15% of *C. elegans *genes [36]. Each operon contains from two to eight genes which are cotranscribed from the same strand as a polycistronic RNA and trans-spliced [36]. It is conceivable that such feature might place a constraint on the plasticity of the worm genome, disfavoring the retention of specific overlap types, such as antiparallel and partial arrangements. Similar genomic constraint has been recently proposed to explain the paucity of duplicated genes in operons [37].

In all genomes except zebrafish, OGCs formed by two genes occurred at a frequency significantly higher than expected (Figure 2A). In addition, OGCs in human, mouse, and fly were mostly formed by antiparallel convergent pairs which overlapped only partially, while in zebrafish and more markedly in worm nested overlaps were preferred (Figures 2B and 2C). However, the results in zebrafish should be taken carefully, since they are probably affected by the poor coverage of the corresponding gene set. Likewise, the annotation of 5' and 3' untranslated regions appears particularly incomplete in worm (Additional file 1), which may contribute to an underestimation of some overlap classes (*i. e*. partial overlap, CDS/UTR and UTR/UTR overlaps, Figure 2). In all species overlaps between genes lying on the same strand and those sharing coding regions are strongly selected against (Figures 2C and 2D). Overlap between UTRs is preferentially retained in all organisms, while the overlap between coding regions and introns is common in zebrafish, drosophila and worm (Figure 2D). The non-random features of observed OGCs suggest that different overlap types are under different selective pressures. The retention of specific overlapping classes might be allowed when it provides selective advantages: in the case of genes on opposite strands the advantage could be represented by antisense regulation. Human, mouse and fly are significantly enriched in overlapping pairs potentially able to form antisense, which include all antiparallel overlaps sharing exons (*H. sapiens *55%, p < 0.001; *M. musculus *58%, p < 0.001; *D. melanogaster *53%, p < 0.001, chi-squared test). This result suggests that, at least in these species, positive selection might act to preserve antisense regulation. It cannot be excluded, however, that part of the positive effect could be a consequence of the negative selection towards parallel and CDS/CDS overlaps.

### Poor evolutionary conservation of OGCs in Metazoa

**Figure 3:**
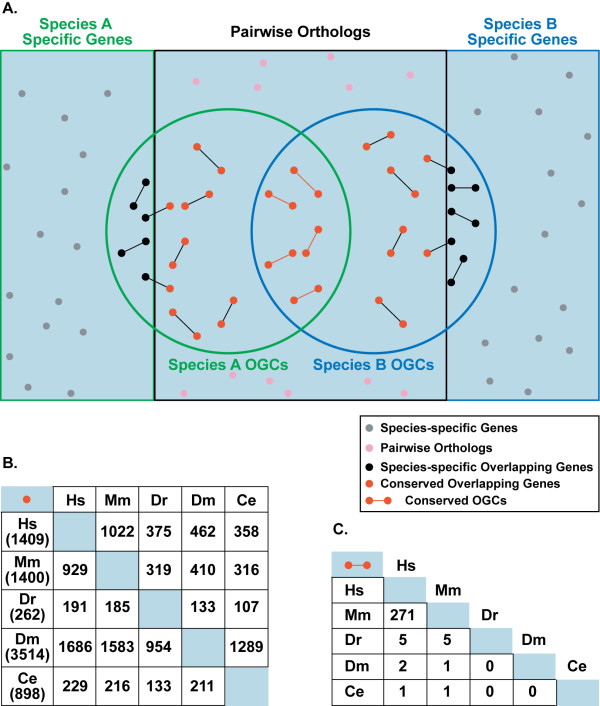
**Conservation of overlapping genes and OGCs within Metazoa**. **A**. Schematic representation of the procedure for detecting the conservation of overlapping genes (red spots) and OGCs (red pairs) between two species. The same pipeline was applied to each pair of species considered in the analysis. **B**. Pairwise conservation of overlapping genes within Metazoans. In the first column, the numbers in brackets represent the total number of overlapping genes for that species. **C**. Pairwise conservation of OGCs within Metazoa.

We next evaluated the conservation of OGCs across metazoan evolution by verifying both the presence of orthologous genes and the overlap conservation. For each pair of analyzed species, we assigned pairwise orthology for all sequence entries, extracted the orthologs involved in OGCs, and verified whether the overlapping arrangement was conserved (Figure 3A). Most overlapping genes in one species had their corresponding orthologs in the others (Figure 3B), but very few overlaps were maintained (Figure 3C). In total, ~40% of human OGCs were also present in mouse -a higher percentage than previous estimates (6.6–17%) [6,13,33,38], but lower than the rate of orthologous genes between the two species (75.6%).

Among OGCs conserved between human and mouse, the antiparallel arrangement was represented the most (~88%), highlighting again the tendency to maintain possible sense-antisense regulation. Interestingly, convergent and nested antiparallel arrangements were significantly enriched in the conserved set (chi-square = 22.47, p = 2.14e-6 and chi-square = 23.55, p = 1.2e-6, respectively), when compared to divergent overlaps (Table 2). This result supports previous observations that 3'-3' (convergent) overlapping pairs are significantly more conserved than 5'5' (divergent) ones, and indicates a prevalent role for 3'UTRs in antisense regulation [14,39].

**Table 2:** Overlapping genes conservation between human and mouse.

	**Total human OG pairs**	**Human-mouse conserved OG pairs**	**Conservation rate (%)**
**OG Pairs**	749	282	37.65
*Partial*	*476*	*172*	*36.13*
partial convergent	328	153	46.65
partial divergent	115	14	12.17
partial parallel	33	5	15.15
*Complete*	*273*	*110*	*40.29*
nested antiparallel	152	79	51.97
nested parallel	75	22	29.33
embedded antiparallel	16	3	18.75
embedded parallel	30	6	20.00

Conservation of overlapping gene (OG) pairs according to their reciprocal arrangement.

Parallel OGCs did not show any significant enrichment in the conserved set (Table 2). Since same-strand overlaps are strongly selected against (Table 1), we investigated whether the ones that are conserved are more likely to be functional. Indeed, we found that several parallel OGCs conserved between human and mouse might be functionally related on the basis of the available literature data (Additional data file 3).

Although the vast majority of overlap is not conserved over long evolutionary distances, we found evidence of few ancient overlaps. Overall, three OGCs were conserved between Ecdysozoa (nematodes and arthropods) and Deuterostomia (vertebrates). Interestingly, the only OGC that is conserved from *C. elegans *to human was lost in arthropods, while two different OGCs are conserved from *D. melanogaster *to human. All of these OGCs are formed of two genes with a nested antiparallel arrangement. One of the two clusters conserved in *D. melanogaster *(Cluster 77, Additional File 2) involves the synapsin (*Syn*) and an inhibitor of metalloproteinase (*Timp*) genes. According to the model proposed for the evolution of the *Syn-Timp *cluster [40], the locus containing the ancestral nested genes has undergone gene duplications and losses in vertebrates, followed by function partitioning among the resulting paralogs. A comparable succession of events is compatible also with the evolution of the only OGC conserved between vertebrates and worm (Cluster 371, Additional File 2). In this case, the ancestral OGC locus seems to have undergone duplication after the split between Protostomia and Deuterostomia, followed by function partitioning among the resulting paralogs (Additional file 4).

The poor evolutionary conservation of gene overlap in Metazoa suggests that its occurrence is species-specific. Such species-specificity was not due to a recent origin of the overlapping genes, as previously suggested [2,13,32]. We found that most overlapping genes in one species had orthologs in the other species, although they did not overlap (Figures 3B and 3C). In addition, 30.2% of human overlapping genes and 25.8% of mouse overlapping genes remained physically adjacent in the compared genome, although the superimposition was lost (see below).

There are examples of functional processes whose poor conservation during evolution is part of their functional role, alternative splicing being the most striking one [41]. Although approximately two-thirds of human genes are alternatively spliced [42], only 10–20% of them conserve the spliced exons in the orthologous genes in mouse [43]. Hence we can propose a species-specific usage of gene overlap similarly to what seems to happen for alternative splicing [41].

### Gene structure modifications associated with overlap formation

In order to infer possible mechanisms for overlap formation, we compared the gene structure (gene length and exon number) of conserved and non-conserved overlapping genes in human and mouse. In particular, we analyzed the gene structure of human and mouse overlapping genes whose orthologs lie adjacent (*i. e*. without any gene between them) but do not overlap in the other genome. We found that 226 human overlapping gene pairs (corresponding to 30.2% of the total) and 171 mouse overlapping gene pairs (25.8% of the total) had orthologs that do not overlap but remain adjacent in the genome of the other species (Table 3). The 226 human overlapping gene pairs were significantly longer (*z*' = 2.53, *p *= 5.7e-3, Mann-Whitney *U*-test [44], Table 3) and had more exons (*z*' = 2.72, *p *= 3.3e-3) than the mouse orthologs, when compared to the set of conserved overlapping genes (Table 3). Similarly, the 171 human orthologs of mouse overlapping gene pairs were shorter (z' = 2.95, p = 1.6e-3) and were formed with fewer exons (z' = 2.28, p = 1.1e-2) than the conserved overlapping pairs. In addition, non-conserved overlapping gene pairs tended to significantly overlap in their UTRs for both human (chi-square = 23.4, p = 1.3e-6) and mouse (chi-square = 24.2, p = 8.9e-7), when compared to the conserved set (Table 3).

**Table 3:** Gene structure comparison between human and mouse.

	**OG Pairs Conserved in Hs and Mm (282)**		**Human OG Pairs Adjacent in Mm (226)**		**Mouse OG Pairs Adjacent in Hs (171)**		**Non-Overlapping Genes**	
	
	**Human**	**Mouse**	**Human**	**Mouse**	**Human**	**Mouse**	**Human (16385)**	**Mouse (15640)**
**Average Gene Length**	68.5 kb	58.6 kb	49.3 kb	31.4 kb	35.5 kb	31.4 kb	55.4 kb	39.6 kb
**Average Exon Number**	12.4	12.2	11.3	10.8	11.45	11.4	10.2	9.0
**UTR Overlap**	174	200	184	-	-	154	-	-
**CDS Overlap**	108	82	42	-	-	16	-	-

The number of overlapping gene pairs in each analyzed dataset is reported in brackets. CDS overlaps refer to the overlapping genes whose CDS coordinates are superimposed, while UTR overlaps refer to those cases where the gene coordinates (calculated from transcript start to transcript end) are superimposed.

The structural analysis of orthologs of human and mouse overlapping genes that remain adjacent but lack the superimposition shows that the overlap formation is frequently associated with an increase in gene size and exon number. We therefore suggest that the overlap between adjacent genes may originate by species-specific acquisition of additional, non-coding exons. In agreement with our results, most of the loci analyzed by the ENCODE consortium were found to possess distal 5' non-coding exons which map into neighboring genes and tend to be tissue- or cell-line-specific [45].

### Expression patterns of overlapping gene pairs

**Figure 4:**
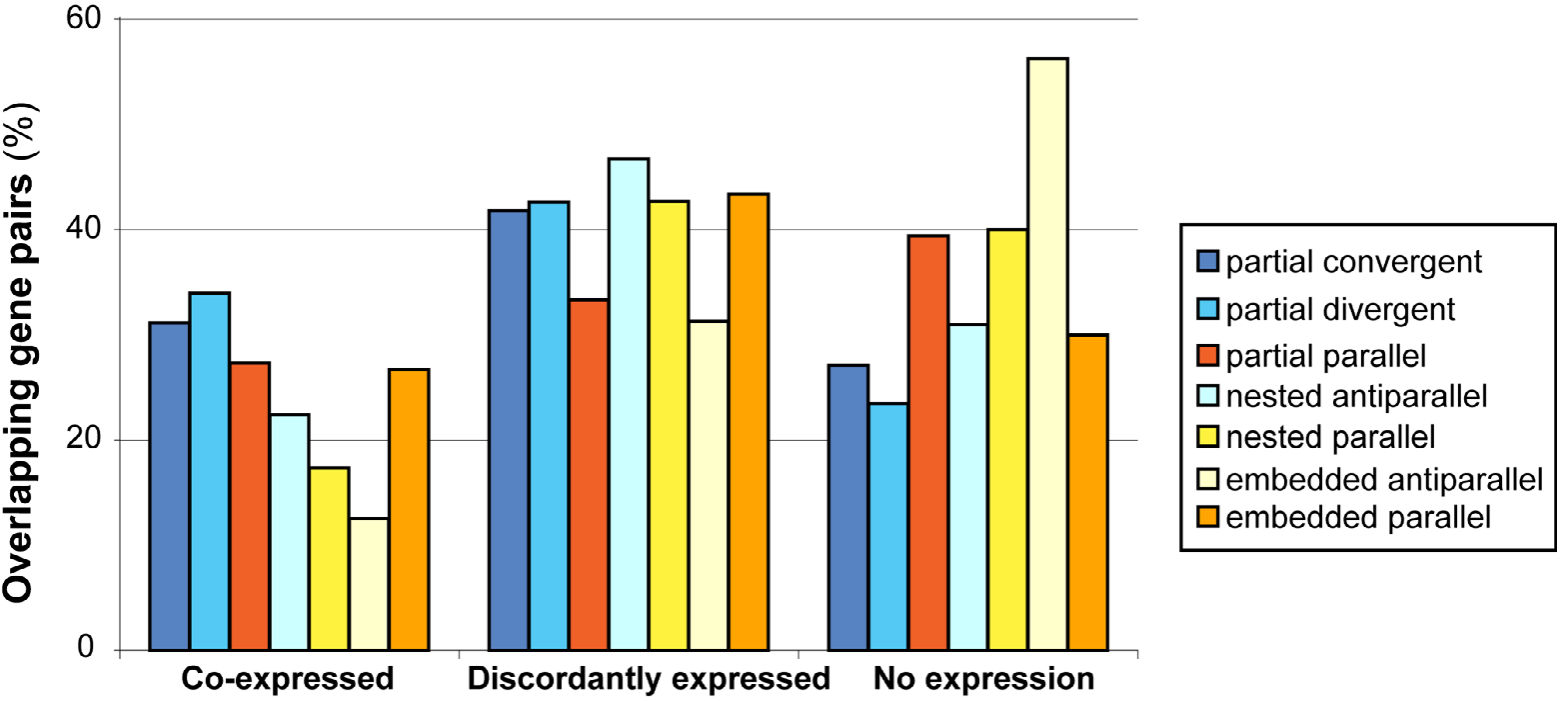
**Analysis of the co-ordinate expression of human overlapping genes**. Expression patterns of human overlapping genes on the basis of their reciprocal arrangement.

In order to evaluate whether the presence of overlap is a mechanism for regulation of gene expression, we used the human OGC dataset to cross-examine a human breast cancer transcriptome obtained by massive pyrosequencing [46]. To be able to detect the expression of transcripts normally expressed at low levels, we used a normalized cDNA library (see Methods). For this reason, our analysis is mostly qualitative and aims to detect the reciprocal expression of genes involved in overlap. Although global gene expression can result quantitatively altered by the tumorous condition, a significant modification in the pattern of reciprocal expression between overlapping genes is unlikely. We defined three patterns of reciprocal expression: co-expression, when both genes were represented in the library; discordant expression, for OG pairs in which expression is observed for only one gene in the pair; and no expression, for OGs whose expression was not detected. Figure 4 shows the frequencies of these three expression patterns in the breast cancer library, by grouping the OG pairs according to the type of overlap.

The observed rate of co-expression in the whole dataset was 27.6%, while the percentage of discordant expressed OGs was 42.5%. Taking into account the overall coverage of known genes in our cDNA library, the co-expression rate is four times higher than expected by the random probability of having any two genes expressed at the same time in the library (7.3%). Therefore, OGs showed a significant tendency to be co-expressed (upper cumulative distribution function, p = 6.7e-102). It should be noted that we obtained significant co-expression even though we removed all sequences mapping to more than one gene in the same cluster (see Methods). Such filtering step likely led to an underestimation of the level of co-expression of overlapping genes, but it did not influence the final result. By contrast, the percentage of discordantly expressed genes is not significantly different from random expectation (upper cumulative distribution function, p = 0.043). Previous studies reported higher co-expression rates, ranging from 35.1% to 44.9% [10,47], with the differences likely due to experimental design (*i. e*. differences in the starting dataset) and in the number of analyzed tissues.

Considering the different overlapping arrangements, we also observed that co-expression was significantly higher for both convergent (chi-square = 4.69, p= 3.03e-2) and divergent OGs (chi-square = 4.28, p= 3.85e-2), when compared to the frequency of the complete overlaps. On the opposite, we observed no statistically significant differences among overlapping arrangements when considering discordantly expressed OGs. Taken together, these results further support the hypothesis that gene overlap might be used to co-ordinate expression of adjacent genes.

## Conclusion

Our work shows for the first time that overlap between protein coding genes, although widespread, is counterselected during Metazoan evolution. We also show that overlap retention does not occur randomly, since it preferentially involves gene pairs lying on opposite DNA strands and sharing non-coding regions. The features of retained OGCs suggest a likely role for overlap in the reciprocal regulation of neighboring genes. The evidence that OGs are significantly co-expressed in the breast cancer transcriptome further supports this hypothesis. In addition, the poor conservation of overlap during evolution, and the fact that formation/loss of the overlapping arrangement is related to changes in gene structure, mostly occurring within non-coding regions, points to this as a species-specific mechanism. As non-coding regions generally have fewer constraints on their primary sequence, the tendency to confine the overlap to non-coding regions may achieve co-regulation without forcing two functional protein-coding genes to co-evolve. We might speculate that this tendency would ultimately result in the evolution of overlapping non-coding transcripts optimized for the regulation of their protein-coding partner.

## Methods

### Overlapping gene detection

The RefSeq cDNA sets [48] for five organisms (*H. sapiens*, *M. musculus*, *D. rerio*, *D. melanogaster*, and *C. elegans*) were downloaded from the UCSC ftp site (RefSeq v.10, March 2005) [49]. We also retrieved mouse cDNAs from the RIKEN database (Fantom 3.0) and the UCSC collection of mouse cDNAs (Mm7 assembly), while for fly and worm we used Flybase (FlyBase r4.2) and Wormbase (WormBase WS140), respectively [50-53].

The genomic position of each sequence was mapped on the corresponding genome by using BLAT [54] (human Build 35; mouse Build 34; zebra fish Zv4; fly Release 4; worm WS120). The pairs of genes whose genomic coordinates partially or totally overlap were extracted and grouped in OGCs. Filters were adopted to avoid (a) splice variants of the same gene, and (b) artifacts due to the position mapping. We considered each pair of cDNAs sharing three or more exons as splice variants of the same gene if more than 20% of the exon number overlapped. In the case of cDNAs with two or less exons, we considered them as splice variants if at least one residue overlapped at the exon level. For each group of predicted splice variants, only the longest gene was taken as gene representative. Artifacts such as the inclusion of the mRNA poly-A in the gene mapping were avoided by excluding all the 3' exons composed of more than 70% of one single nucleotide.

### Statistical null model for the overlap formation

For all five species analyzed, the gene positions of the unique gene sets were randomly reassigned within the corresponding chromosomes with no constraints in the type of overlaps, the reciprocal arrangement, and the number of genes per cluster. The analysis was repeated for 10 rounds and the resulting number of overlapping genes, overlapping gene pairs, and overlapping gene clusters were counted at each round. The average number was considered for comparison with the observed dataset. Features of the OGCs, such as the reciprocal arrangement, the component distribution and the type of overlapping region were also analyzed.

The fraction of overlaps that results in sense/antisense complementarity at the mRNA level were calculated by extracting all overlap that occur on opposite strands and involve exons of both genes. The statistical significance of the difference between the observed and the random set was assessed by applying a chi-squared test (degree of freedom = 1) to the resulting 2 × 2 contingency matrix [44].

### Benchmark

To test the specificity of the data produced, we performed a manual analysis of the *D. rerio *dataset (108 OGCs). No obvious false positive due to the methodology could be found. The sensitivity of our method was assessed by benchmarking the derived set against an extensive collection of overlapping genes previously reported. We included 8 independent large-scale screenings of human antisense transcripts/nested genes [9,13,27,29,30,55-57] and about 100 experimental studies on specific overlapping gene pairs (Additional files 5 and 6). OGCs reported in the literature with no match in our dataset were checked manually. The main reasons for the lack of coverage were due to the selection criteria (*i. e*. we deliberately excluded pseudogenes or non-coding RNAs which were instead included in some large-scale screenings). Only 5 cases were found to be false negatives, giving an estimate specificity of 99%.

### Orthology assignment

The orthology relationships between the overlapping genes in the five analyzed species were assessed by using a two-step procedure (Figure 3A). First, for all pairs of species we carried out all-against-all tBLASTx [58] between the corresponding cDNA sets. The best reciprocal hits between two species were assigned as orthologous genes. Secondly, we derived orthologous overlapping genes by extracting all overlapping genes conserved between each pair of species.

### Gene structure analysis

We compared the gene structure of the conserved OGCs between human and mouse with human and mouse overlapping genes whose orthologs do not overlap but are adjacent in the genome of the other species. The first set (conserved overlapping genes between human and mouse; the first column in Table 3) was composed of 282 pairs of overlapping genes, while the second (overlapping in human but adjacent in mouse chromosomes; the second column in Table 3), and the third (overlapping in mouse but adjacent in human chromosomes; the third column in Table 3) were composed of 226 and 171 gene pairs, respectively. For each gene, we measured the gene length, defined as the genomic coordinates on the corresponding chromosome, and the exon numbers, as derived from the BLAT output. Using the Mann-Whitney U-test [44] we compared gene length difference between the first and the second sets, and between the first and the third sets to assess the statistical significance of the difference in gene structure.

We also analyzed the feature of the region (UTR or coding) involved in the overlap for all OGCs in the 3 sets, by counting the number of detectable overlaps after removing the UTRs. In this case, the statistical significance of the difference between the first and the second sets and the first and the third set were assessed by applying a chi-squared test (degree of freedom = 1) to the resulting 2 × 2 contingency matrix [44].

### Analysis of OGC expression in breast cancer

cDNA was obtained from polyadenylated breast cancer RNA (purity 85–90%). cDNA was normalized after reverse transcription to obtain a balanced mix of low and high abundance mRNA, as previously described [59]. 2.1 micrograms of normalized, double-strand cDNA were then converted to a single strand library using the 454 protocol [46]. Two independent cDNA libraries were generated with an average length per sequence read of 100 and 200 nt, respectively. A total of 198,658 non-redundant sequence reads, according to NCBI non-redundant database, were sequenced from each breast cancer cDNA library. The entire library was mapped against the 249,953 sequences of the human "all_mrna" transcript dataset from the UCSC human genome. A total of 37,774 reads corresponding to a specific cDNA and its related isoforms was identified (requiring blat perfect matches, 95% of the read covered by alignment). The reads were then aligned to the human RefSeq cDNA dataset from UCSC (25,922 sequences) requiring perfect coverage. 9,082 distinct matches were finally obtained, which were used for the subsequent calculations.

Reads-to-gene assignment was performed by blasting the nucleotide sequences of all OGs to the library. Only reads showing 100% identity with a transcript were used in the analyses. To ensure the 454 sequences were unambiguously matched to the assigned transcript, we removed reads mapped to more than one locus. Since the 454 sequencing process does not involve *in-vivo *cloning and the cDNA is subjected to nebulization, in the deriving library it is not possible to assign the strand when the two transcripts overlap. Thus, we removed all sequence reads mapping to more than one gene within the same cluster. In total, 36 out of 3701 reads were removed, corresponding to an estimated loss of 0.9%, which likely did not create a significant bias.

The statistical significance for the enrichment of co-expression in overlapping gene pairs was evaluated by an upper cumulative distribution function.

## Authors' contributions

GS contributed to the study concept and design (Gene structure analysis), the data collection (Features of human overlapping genes, Benchmark), the analysis and interpretation of the data, and drafted the manuscript. MS was involved in the study design (Statistical null model of overlap formation), the data collection (Orthology assignment), the analysis and interpretation of the data, and provided his statistical expertise. PP built the cDNA library. SB contributed to the data interpretation as well as to the drafting of the manuscript. AG and ER did the pyrosequencing and primary sequence analysis of the cDNA library. PB and MT provided critical revision of the manuscript for important intellectual content. FDC contributed to the study concept and design, the analysis and interpretation of the data, the drafting of the manuscript, and supervised the entire study.

## Supplementary Material

Additional file 1 Features of the unique RefSeq genes used for the analysis. Click here for file

Additional file 2 **Datasets of overlapping gene clusters in five metazoan genomes**. The file is formatted with one worksheet for each species analyzed. For each dataset the cluster number, the number of component and the list of RefSeq Accession numbers of all components are reported. Click here for file

Additional file 3 **Parallel overlaps conserved between human and mouse**. We manually reviewed the main literature on the genes involved in parallel OGCs conserved between human and mouse to look for possible functional links. We signed as 'Not Known' the cases where either the transcripts correspond to not yet annotated genes, or no functional link can be derived from the available literature. Click here for file

Additional file 4 Phylogenetic analysis of the OGC conserved between nematodes and vertebrates. Click here for file

Additional file 5 **Literature overview of human overlapping genes**. The numbering for the literature references refers to Additional file 6. Click here for file

Additional file 6 **Additional bibliographic references**. Document providing all the literature references cited in the Additional data files. Click here for file
