# Supplementary material for: Non-random retention of protein-coding overlapping genes in Metazoa
Source: BMC Genomics. 2008 Apr 16;9:174. doi: 10.1186/1471-2164-9-174 (PMC2330155; doi:10.1186/1471-2164-9-174)
Supplement: Additional file 1 — Features of the unique RefSeq genes used for the analysis. [file 1471-2164-9-174-S1.pdf]

Additional file 1. Features of the unique RefSeq genes used for analysis.

Species	Chromosomes (Mb)	Genes[1] (Mb)	Gene Density[2]	CDSs[1] (Mb)	Introns (Mb)	Intron per Gene (%)	UTRs[1] (Mb)	UTR per Gene (%)
Hs	3950.0	978.6	24.8	840.6	931.1	95.1	138.0	14.1
Mm	3165.0	684.7	21.6	569.0	645.5	94.3	115.7	16.9
Dr	1589.3	131.2	8.3	110.2	120.7	92.0	20.9	15.9
Dm	129.3	66.7	51.6	48.5	40.1	60.1	18.1	27.1
Ce	100.3	48.7	48.6	47.1	25.8	53.0	1.5	3.1

[1]The length does not correspond to the length of the corresponding transcript but to the length of the genomic sequence including introns.

[2]The gene density is calculated as geneLength/chrLength.

Abbreviations: *Hs*, Homo sapiens; *Mm*, Mus musculus; *Dr*, Danio rerio; *Dm*, Drosophila melanogaster; *Ce*, Caenorhabditis elegans.
