# Supplementary material for: Non-random retention of protein-coding overlapping genes in Metazoa
Source: BMC Genomics. 2008 Apr 16;9:174. doi: 10.1186/1471-2164-9-174 (PMC2330155; doi:10.1186/1471-2164-9-174)
Supplement: Additional file 4 — Phylogenetic analysis of the OGC conserved between nematodes and vertebrates. [file 1471-2164-9-174-S4.pdf]

Additional file 4. Phylogenetic analysis of the OGC conserved between nematodes and vertebrates. A. In nematodes, the embedded gene (*Mab-21*) has been shown to regulate the formation of sensory organs during the worm tail development [104]. The host gene is the ortholog of two vertebrate genes, the neurobeachin (*Nbea*), and the lipopolysaccharide-inducible gene (*Lrba*). These genes code for two kinase anchor proteins, and the first is disrupted in idiopathic autism [105]. The duplication of the locus containing the nested genes happened after the split between Protostomia and Deuterostomia. The longest and most complex gene is always the *Nbea/Lrba* ortholog, while *Mab2-l1/Mab2-l2* is the smallest one, although the deuterostomal paralogs *Nbea* and *Lrba* acquired a more complex gene structure, with the insertion of large introns. Different scale bars have been used for fly and worm, as the corresponding genes are much shorter than the vertebrate ones. In *D.melanogaster* the genes are not overlapping but they both localize on chromosome X. The distance between them is around 740 kb. The branches corresponding to the two Protostomia have been left unresolved at the root of the tree, because of their dubious reciprocal phylogenetic relationship. For the tree reconstruction, only the proteins encoded by the metazoan orthologs of *Mab2-l1* and *Mab2-l2* were used. Moreover, the sequence of ciona was excluded from the analysis, as both genes are incomplete. The multiple alignment was built using MUSCLE [106], with the maximum number of iterations set to 100. The poorly aligned and divergent regions were eliminated using Gblocks [107], with the maximum number of contiguous non-conserved positions set to 8, the minimum length of a block set to 2, and allowing intermediate gap positions. The Maximum Likelihood (ML) phylogenetic tree was inferred using PHYML [108], and applying the JTT matrix. The applied model of evolution assumed the presence of two classes of sites, one invariable and the other free to change. The rate variation across

these sites was assumed to follow a gamma shape distribution calculated using a discrete approximation with four categories of sites. Support for the hypothesized relationships was assessed using 1000 bootstrap replicates.

B. For human, mouse, fly and worm the sequences of *Nbea*/*Lrba* and *Mab2-l1*/*Mab2-l2* present in the corresponding protein databases were used. For rat, chicken, zebrafish and ciona the protein sequences were predicted manually. The human ortholog was used as a query for stand-alone tBLASTn [109] in the corresponding genome sequence. Once the gene regions were detected, the gene structure and the correct protein sequences were assigned by using GeneWise [110]. *Lrba*/*Mab21L1* correspond to human Cluster371, while *Nbea*/*Mab21L2* correspond to human Cluster449 (see Additional file 2). Cluster449 does not appear among the human OGCs conserved in mouse, as CAC18811 is not present in the RefSeq version used in the analysis.

A Phylogenetic tree

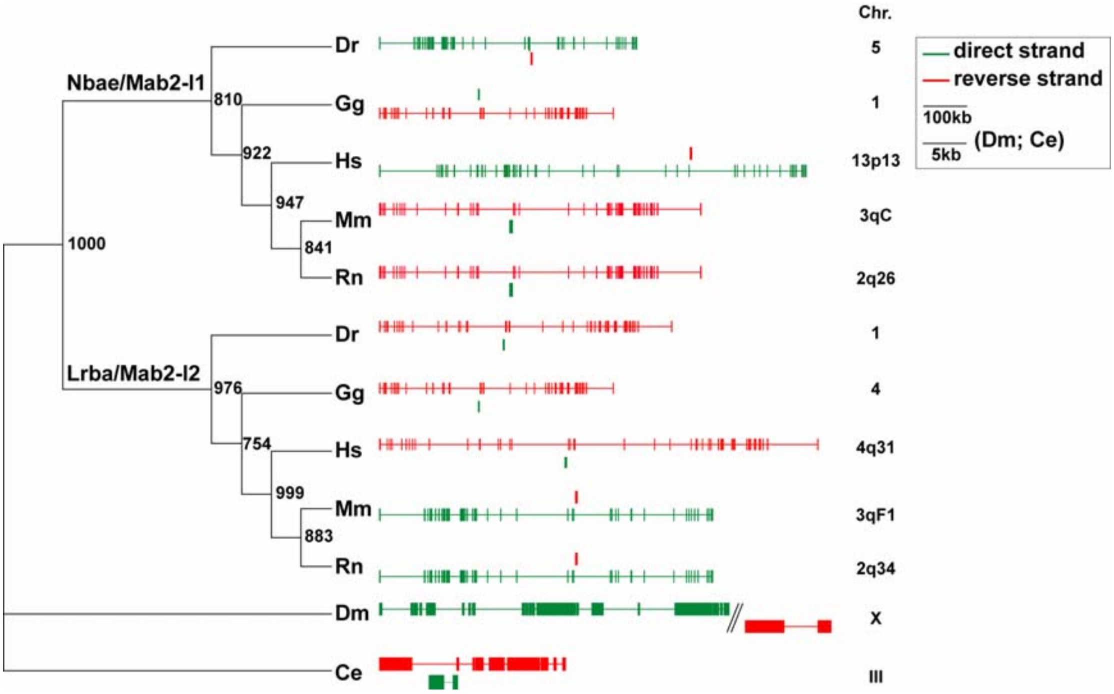

B Metazoan orthologs of *Nbea*/*Lrba* and *Mab2-l1*/*Mab2-l2* encoded proteins

Protein	Human	Mouse	Rat	Chicken	Zebrafish	Fly	Worm
**LRBA**	NP_006717	NP_109620	chr2_lrba.fa	chr1_lrba	chr5_lrba	CG6775	F10F2.1
**NBEA**	NP_056493	CAC18811	chr2_nbea.fa	chr1_nbea	chr5_nbea		
**MAB21-L1**	NP_005575	NP_034880	chr2_mab2l1	chr1_mab2l1	chr5_mab2l1	CG4746	F35G12.6
**MAB21-L2**	NP_006430	NP_035969	chr2_mab2l2	chr1_mab2l2	chr5_mab2l2		
