# Supplementary material for: Non-random retention of protein-coding overlapping genes in Metazoa
Source: BMC Genomics. 2008 Apr 16;9:174. doi: 10.1186/1471-2164-9-174 (PMC2330155; doi:10.1186/1471-2164-9-174)
Supplement: Additional file 6 — Additional bibliographic references. Document providing all the literature references cited in the Additional data files. [file 1471-2164-9-174-S6.pdf]

REFERENCES

1. Shibuya K, Obayashi I, Asakawa S, Minoshima S, Kudoh J, et al. (2004) A cluster of 21 keratin-associated protein genes within introns of another gene on human chromosome 21q22.3. Genomics 83: 679-693.

2. Veeramachaneni V, Makalowski W, Galdzicki M, Sood R, Makalowska I (2004) Mammalian overlapping genes: the comparative perspective. Genome Res 14: 280-286.

3. Quere R, Manchon L, Lejeune M, Clement O, Pierrat F, et al. (2004) Mining SAGE data allows large-scale, sensitive screening of antisense transcript expression. Nucleic Acids Res 32: e163.

4. Viskochil D, Cawthon R, O'Connell P, Xu GF, Stevens J, et al. (1991) The gene encoding the oligodendrocyte-myelin glycoprotein is embedded within the neurofibromatosis type 1 gene. Mol Cell Biol 11: 906-912.

5. Yu P, Ma D, Xu M (2005) Nested genes in the human genome. Genomics 86: 414-422.

6. Kasper G, Taudien S, Staub E, Mennerich D, Rieder M, et al. (2002) Different structural organization of the encephalopsin gene in man and mouse. Gene 295: 27-32.

7. Halford S, Freedman MS, Bellingham J, Inglis SL, Poopalasundaram S, et al. (2001) Characterization of a novel human opsin gene with wide tissue expression and identification of embedded and flanking genes on chromosome 1q43. Genomics 72: 203-208.

8. Chen J, Sun M, Kent WJ, Huang X, Xie H, et al. (2004) Over 20% of human transcripts might form sense-antisense pairs. Nucleic Acids Res 32: 4812-4820.

9. Trinklein ND, Aldred SF, Hartman SJ, Schroeder DI, Otillar RP, et al. (2004) An abundance of bidirectional promoters in the human genome. Genome Res 14: 62-66.

10. Li B, Zhuang L, Trueb B (2004) Zyxin interacts with the SH3 domains of the cytoskeletal proteins LIM-nebulette and Lasp-1. J Biol Chem 279: 20401-20410.

11. Fahey ME, Moore TF, Higgins DG (2002) Overlapping antisense transcription in the human genome. Comp Funct Genomics 3: 244-253.

12. Lehner B, Williams G, Campbell RD, Sanderson CM (2002) Antisense transcripts in the human genome. Trends Genet 18: 63-65.

13. Scherer SW, Cheung J, MacDonald JR, Osborne LR, Nakabayashi K, et al. (2003) Human chromosome 7: DNA sequence and biology. Science 300: 767-772.

14. Holm SJ, Carlen LM, Mallbris L, Stahle-Backdahl M, O'Brien KP (2003) Polymorphisms in the SEEK1 and SPR1 genes on 6p21.3 associate with psoriasis in the Swedish population. Exp Dermatol 12: 435-444.

15. Tanaka H, Kohroki J, Iguchi N, Onishi M, Nishimune Y (2002) Cloning and characterization of a human orthologue of testis-specific succinyl CoA: 3-oxo acid CoA transferase (Scot-t) cDNA. Mol Hum Reprod 8: 16-23.

16. Ubeda M, Schmitt-Ney M, Ferrer J, Habener JF (1999) CHOP/GADD153 and methionyl-tRNA synthetase (MetRS) genes overlap in a conserved region that controls mRNA stability. Biochem Biophys Res Commun 262: 31-38.

17. Levinson B, Kenwrick S, Lakich D, Hammonds G, Jr., Gitschier J (1990) A transcribed gene in an intron of the human factor VIII gene. Genomics 7: 1-11.

18. Levinson B, Kenwrick S, Gamel P, Fisher K, Gitschier J (1992) Evidence for a third transcript from the human factor VIII gene. Genomics 14: 585-589.

19. Dent AL, Yewdell J, Puvion-Dutilleul F, Koken MH, de The H, et al. (1996) LYSP100-associated nuclear domains (LANDs): description of a new class of subnuclear structures and their relationship to PML nuclear bodies. Blood 88: 1423-1426.

20. Herzog H, Darby K, Hort YJ, Shine J (1996) Intron 17 of the human retinoblastoma susceptibility gene encodes an actively transcribed G protein-coupled receptor gene. Genome Res 6: 858-861.

21. Ralph SJ, Thomas ML, Morton CC, Trowbridge IS (1987) Structural variants of human T200 glycoprotein (leukocyte-common antigen). Embo J 6: 1251-1257.

22. Yu WP, Brenner S, Venkatesh B (2003) Duplication, degeneration and subfunctionalization of the nested synapsin-Timp genes in Fugu. Trends Genet 19: 180-183.

23. Strausberg RL, Feingold EA, Grouse LH, Derge JG, Klausner RD, et al. (2002) Generation and initial analysis of more than 15,000 full-length human and mouse cDNA sequences. Proc Natl Acad Sci U S A 99: 16899-16903.

24. Svaren J, Apel ED, Simburger KS, Jenkins NA, Gilbert DJ, et al. (1997) The Nab2 and Stat6 genes share a common transcription termination region. Genomics 41: 33-39.

25. Larsen F, Solheim J, Kristensen T, Kolsto AB, Prydz H (1993) A tight cluster of five unrelated human genes on chromosome 16q22.1. Hum Mol Genet 2: 1589-1595.

26. Lauren J, Airaksinen MS, Saarma M, Timmusk T (2003) A novel gene family encoding leucine-rich repeat transmembrane proteins differentially expressed in the nervous system. Genomics 81: 411-421.

27. Conrad C, Vianna C, Freeman M, Davies P (2002) A polymorphic gene nested within an intron of the tau gene: implications for Alzheimer's disease. Proc Natl Acad Sci U S A 99: 7751-7756.

28. Yelin R, Dahary D, Sorek R, Levanon EY, Goldstein O, et al. (2003) Widespread occurrence of antisense transcription in the human genome. Nat Biotechnol 21: 379-386.

29. Ohinata Y, Sutou S, Kondo M, Takahashi T, Mitsui Y (2002) Male-enhanced antigen-1 gene flanked by two overlapping genes is expressed in late spermatogenesis. Biol Reprod 67: 1824-1831.

30. van Duin M, van Den Tol J, Hoeijmakers JH, Bootsma D, Rupp IP, et al. (1989) Conserved pattern of antisense overlapping transcription in the homologous human ERCC-1 and yeast RAD10 DNA repair gene regions. Mol Cell Biol 9: 1794-1798.

31. Whitehead CM, Winkfein RJ, Fritzler MJ, Rattner JB (1997) ASE-1: a novel protein of the fibrillar centres of the nucleolus and nucleolus organizer region of mitotic chromosomes. Chromosoma 106: 493-502.

32. Shimmoto M, Nakahori Y, Matsushita I, Shinka T, Kuroki Y, et al. (1996) A human protective protein gene partially overlaps the gene encoding phospholipid

transfer protein on the complementary strand of DNA. Biochem Biophys Res Commun 220: 802-806.

33. Stover C, Gradl G, Jentsch I, Speicher MR, Wieser R, et al. (2001) cDNA cloning, chromosome assignment, and genomic structure of a human gene encoding a novel member of the RBM family. Cytogenet Cell Genet 92: 225-230.

34. Bejanin S, Cervini R, Mallet J, Berrard S (1994) A unique gene organization for two cholinergic markers, choline acetyltransferase and a putative vesicular transporter of acetylcholine. J Biol Chem 269: 21944-21947.

35. Dear TN, Meier NT, Hunn M, Boehm T (2000) Gene structure, chromosomal localization, and expression pattern of Capn12, a new member of the calpain large subunit gene family. Genomics 68: 152-160.

36. Kawabata Y, Hata S, Ono Y, Ito Y, Suzuki K, et al. (2003) Newly identified exons encoding novel variants of p94/calpain 3 are expressed ubiquitously and overlap the alpha-glucosidase C gene. FEBS Lett 555: 623-630.

37. Wong JC, Alon N, Norga K, Kruyt FA, Youssoufian H, et al. (2000) Cloning and analysis of the mouse Fanconi anemia group A cDNA and an overlapping penta zinc finger cDNA. Genomics 67: 273-283.

38. Gallagher PG, Forget BG (1998) An alternate promoter directs expression of a truncated, muscle-specific isoform of the human ankyrin 1 gene. J Biol Chem 273: 1339-1348.

39. Higgins JM (2001) The Haspin gene: location in an intron of the integrin alphaE gene, associated transcription of an integrin alphaE-derived RNA and expression in diploid as well as haploid cells. Gene 267: 55-69.

40. Faurholm B, Millar RP, Katz AA (2001) The genes encoding the type II gonadotropin-releasing hormone receptor and the ribonucleoprotein RBM8A in humans overlap in two genomic loci. Genomics 78: 15-18.

41. Okubo K, Mitani H, Naruse K, Kondo M, Shima A, et al. (2002) Conserved physical linkage of GnRH-R and RBM8 in the medaka and human genomes. Biochem Biophys Res Commun 293: 327-331.

42. Fujii H, Shimada T (1989) Isolation and characterization of cDNA clones derived from the divergently transcribed gene in the region upstream from the human dihydrofolate reductase gene. J Biol Chem 264: 10057-10064.

43. Travers MT, Cambot M, Kennedy HT, Lenoir GM, Barber MC, et al. (2005) Asymmetric expression of transcripts derived from the shared promoter between the divergently oriented ACACA and TADA2L genes. Genomics 85: 71-84.

44. Bera TK, Das S, Maeda H, Beers R, Wolfgang CD, et al. (2004) NGEP, a gene encoding a membrane protein detected only in prostate cancer and normal prostate. Proc Natl Acad Sci U S A 101: 3059-3064.

45. Robb GB, Carson AR, Tai SC, Fish JE, Singh S, et al. (2004) Post-transcriptional regulation of endothelial nitric-oxide synthase by an overlapping antisense mRNA transcript. J Biol Chem 279: 37982-37996.

46. Lafreniere RG, MacDonald ML, Dube MP, MacFarlane J, O'Driscoll M, et al. (2004) Identification of a novel gene (HSN2) causing hereditary sensory and

autonomic neuropathy type II through the Study of Canadian Genetic Isolates. Am J Hum Genet 74: 1064-1073.

47. Chatterjee TK, Fisher RA (2000) Novel alternative splicing and nuclear localization of human RGS12 gene products. J Biol Chem 275: 29660-29671.

48. Cheng J, Kapranov P, Drenkow J, Dike S, Brubaker S, et al. (2005) Transcriptional maps of 10 human chromosomes at 5-nucleotide resolution. Science 308: 1149-1154.

49. Le Poole IC, Sarangarajan R, Zhao Y, Stennett LS, Brown TL, et al. (2001) 'VIT1', a novel gene associated with vitiligo. Pigment Cell Res 14: 475-484.

50. Katoh M, Yazaki Y, Sugimura T, Terada M (1993) c-erbB3 gene encodes secreted as well as transmembrane receptor tyrosine kinase. Biochem Biophys Res Commun 192: 1189-1197.

51. Lee H, Maihle NJ (1998) Isolation and characterization of four alternate c-erbB3 transcripts expressed in ovarian carcinoma-derived cell lines and normal human tissues. Oncogene 16: 3243-3252.

52. Adachi N, Lieber MR (2002) Bidirectional gene organization: a common architectural feature of the human genome. Cell 109: 807-809.

53. Fujiwara N, Matsuo T, Ohtsuki H (2003) Protein expression, genomic structure, and polymorphisms of oculomedin. Ophthalmic Genet 24: 141-151.

54. West AB, Lockhart PJ, O'Farell C, Farrer MJ (2003) Identification of a novel gene linked to parkin via a bi-directional promoter. J Mol Biol 326: 11-19.

55. Funke B, Pandita RK, Morrow BE (2001) Isolation and characterization of a novel gene containing WD40 repeats from the region deleted in velo-cardio-facial/DiGeorge syndrome on chromosome 22q11. Genomics 73: 264-271.

56. Bates EE, Kissenpfennig A, Peronne C, Mattei MG, Fossiez F, et al. (2000) The mouse and human IGSF6 (DORA) genes map to the inflammatory bowel disease 1 locus and are embedded in an intron of a gene of unknown function. Immunogenetics 52: 112-120.

57. Gardiner K, Slavov D, Bechtel L, Davisson M (2002) Annotation of human chromosome 21 for relevance to Down syndrome: gene structure and expression analysis. Genomics 79: 833-843.

58. Dan I, Watanabe NM, Kajikawa E, Ishida T, Pandey A, et al. (2002) Overlapping of MINK and CHRNE gene loci in the course of mammalian evolution. Nucleic Acids Res 30: 2906-2910.

59. Gray TA, Azama K, Whitmore K, Min A, Abe S, et al. (2001) Phylogenetic conservation of the makorin-2 gene, encoding a multiple zinc-finger protein, antisense to the RAF1 proto-oncogene. Genomics 77: 119-126.

60. Kiss H, Darai E, Kiss C, Kost-Alimova M, Klein G, et al. (2002) Comparative human/murine sequence analysis of the common eliminated region 1 from human 3p21.3. Mamm Genome 13: 646-655.

61. Roginski RS, Mohan Raj BK, Birditt B, Rowen L (2004) The human GRINL1A gene defines a complex transcription unit, an unusual form of gene organization in eukaryotes. Genomics 84: 265-276.

62. Prakash SK, Paylor R, Jenna S, Lamarche-Vane N, Armstrong DL, et al. (2000) Functional analysis of ARHGAP6, a novel GTPase-activating protein for RhoA. Hum Mol Genet 9: 477-488.

63. Petrukhin K, Koisti MJ, Bakall B, Li W, Xie G, et al. (1998) Identification of the gene responsible for Best macular dystrophy. Nat Genet 19: 241-247.

64. Bai C, Connolly B, Metzker ML, Hilliard CA, Liu X, et al. (2000) Overexpression of M68/DcR3 in human gastrointestinal tract tumors independent of gene amplification and its location in a four-gene cluster. Proc Natl Acad Sci U S A 97: 1230-1235.

65. Semple JI, Ribas G, Hillyard G, Brown SE, Sanderson CM, et al. (2003) A novel gene encoding a coiled-coil mitochondrial protein located at the telomeric end of the human MHC Class III region. Gene 314: 41-54.

66. Melchior C, Kreis S, Janji B, Kieffer N (2002) Promoter characterization and genomic organization of the gene encoding integrin-linked kinase 1. Biochim Biophys Acta 1575: 117-122.

67. Simeone A, Pannese M, Acampora D, D'Esposito M, Boncinelli E (1988) At least three human homeoboxes on chromosome 12 belong to the same transcription unit. Nucleic Acids Res 16: 5379-5390.

68. Hastings ML, Ingle HA, Lazar MA, Munroe SH (2000) Post-transcriptional regulation of thyroid hormone receptor expression by cis-acting sequences and a naturally occurring antisense RNA. J Biol Chem 275: 11507-11513.

69. Laudet V, Begue A, Henry-Duthoit C, Joubel A, Martin P, et al. (1991) Genomic organization of the human thyroid hormone receptor alpha (c-erbA-1) gene. Nucleic Acids Res 19: 1105-1112.

70. Weisenberger DJ, Velicescu M, Preciado-Lopez MA, Gonzales FA, Tsai YC, et al. (2002) Identification and characterization of alternatively spliced variants of DNA methyltransferase 3a in mammalian cells. Gene 298: 91-99.

71. Castermans D, Wilquet V, Parthoens E, Huysmans C, Steyaert J, et al. (2003) The neurobeachin gene is disrupted by a translocation in a patient with idiopathic autism. J Med Genet 40: 352-356.

72. Poulin F, Brueschke A, Sonenberg N (2003) Gene fusion and overlapping reading frames in the mammalian genes for 4E-BP3 and MASK. J Biol Chem 278: 52290-52297.

73. Shintani S, O'HUigin C, Toyosawa S, Michalova V, Klein J (1999) Origin of gene overlap: the case of TCP1 and ACAT2. Genetics 152: 743-754.

74. Bailleul B, Akerblom I, Strosberg AD (1997) The leptin receptor promoter controls expression of a second distinct protein. Nucleic Acids Res 25: 2752-2758.

75. Rampersad V, Elliott CE, Nutt SL, Foldes RL, Kamboj RK (1994) Human glutamate receptor hGluR3 flip and flop isoforms: cloning and sequencing of the cDNAs and primary structure of the proteins. Biochim Biophys Acta 1219: 563-566.

76. Watterson DM, Schavocky JP, Guo L, Weiss C, Chlenski A, et al. (1999) Analysis of the kinase-related protein gene found at human chromosome 3q21 in a multi-gene cluster: organization, expression, alternative splicing, and polymorphic marker. J Cell Biochem 75: 481-491.

77. Dunham I, Shimizu N, Roe BA, Chissoe S, Hunt AR, et al. (1999) The DNA sequence of human chromosome 22. Nature 402: 489-495.

78. Whitmore SA, Settasatian C, Crawford J, Lower KM, McCallum B, et al. (1998) Characterization and screening for mutations of the growth arrest-specific 11 (GAS11) and C16orf3 genes at 16q24.3 in breast cancer. Genomics 52: 325-331.

79. Rollinger-Holzinger I, Eibl B, Pauly M, Griesser U, Hentges F, et al. (2000) LST1: a gene with extensive alternative splicing and immunomodulatory function. J Immunol 164: 3169-3176.

80. Vuoristo JT, Berrettini WH, Ala-Kokko L (2001) C18orf2, a novel, highly conserved intronless gene within intron 5 of the GNAL gene on chromosome 18p11. Cytogenet Cell Genet 93: 19-22.

81. Plougastel B, Trowsdale J (1998) Sequence analysis of a 62-kb region overlapping the human KLRC cluster of genes. Genomics 49: 193-199.

82. Glienke J, Sobanov Y, Brostjan C, Steffens C, Nguyen C, et al. (1998) The genomic organization of NKG2C, E, F, and D receptor genes in the human natural killer gene complex. Immunogenetics 48: 163-173.

83. Lobley A, Pierron V, Reynolds L, Allen L, Michalovich D (2003) Identification of human and mouse CatSper3 and CatSper4 genes: characterisation of a common interaction domain and evidence for expression in testis. Reprod Biol Endocrinol 1: 53.

84. Meng X, Liu J, Shen Z (2003) Genomic structure of the human BCCIP gene and its expression in cancer. Gene 302: 139-146.

85. Daniels RJ, Peden JF, Lloyd C, Horsley SW, Clark K, et al. (2001) Sequence, structure and pathology of the fully annotated terminal 2 Mb of the short arm of human chromosome 16. Hum Mol Genet 10: 339-352.

86. Meijerink PH, Yanakiev P, Zorn I, Grierson AJ, Bikker H, et al. (1998) The gene for the human Src-like adaptor protein (hSLAP) is located within the 64-kb intron of the thyroglobulin gene. Eur J Biochem 254: 297-303.

87. Van Eynde A, Perez-Callejon E, Schoenmakers E, Jacquemin M, Stalmans W, et al. (1999) Organization and alternate splice products of the gene encoding nuclear inhibitor of protein phosphatase-1 (NIPP-1). Eur J Biochem 261: 291-300.

88. Laitinen T, Polvi A, Rydman P, Vendelin J, Pulkkinen V, et al. (2004) Characterization of a common susceptibility locus for asthma-related traits. Science 304: 300-304.

89. Bohren KM, Nadkarni V, Song JH, Gabbay KH, Owerbach D (2004) A M55V polymorphism in a novel SUMO gene (SUMO-4) differentially activates heat shock transcription factors and is associated with susceptibility to type I diabetes mellitus. J Biol Chem 279: 27233-27238.

90. Guo D, Li M, Zhang Y, Yang P, Eckenrode S, et al. (2004) A functional variant of SUMO4, a new I kappa B alpha modifier, is associated with type 1 diabetes. Nat Genet 36: 837-841.

91. Sharpless NE, DePinho RA (1999) The INK4A/ARF locus and its two gene products. Curr Opin Genet Dev 9: 22-30.

92. Kim J, Bergmann A, Stubbs L (2000) Exon sharing of a novel human zinc-finger gene, ZIM2, and paternally expressed gene 3 (PEG3). Genomics 64: 114-118.

93. Duga S, Solda G, Asselta R, Bonati MT, Dalpra L, et al. (2001) Characterization of the genomic structure of the human neuronal nicotinic acetylcholine receptor CHRNA5/A3/B4 gene cluster and identification of novel intragenic polymorphisms. J Hum Genet 46: 640-648.

94. Herzog H, Darby K, Ball H, Hort Y, Beck-Sickinger A, et al. (1997) Overlapping gene structure of the human neuropeptide Y receptor subtypes Y1 and Y5 suggests coordinate transcriptional regulation. Genomics 41: 315-319.

95. Wende H, Volz A, Ziegler A (2000) Extensive gene duplications and a large inversion characterize the human leukocyte receptor cluster. Immunogenetics 51: 703-713.

96. Verde I, Pahlke G, Salanova M, Zhang G, Wang S, et al. (2001) Myomegalin is a novel protein of the golgi/centrosome that interacts with a cyclic nucleotide phosphodiesterase. J Biol Chem 276: 11189-11198.

97. Martin AM, Freitas EM, Witt CS, Christiansen FT. (2000) The genomic organization and evolution of the natural killer immunoglobulin-like receptor (KIR) gene cluster. Immunogenetics. 51:268-80.

98. Gonzalez A, Saez ME, Aragon MJ, Galan JJ, Vettori P, et al. (2006) Specific haplotypes of the CALPAIN-5 gene are associated with polycystic ovary syndrome. Hum Reprod. 21:943-51.

99. Seemann S, Hainaut P. (2005) Roles of thioredoxin reductase 1 and APE/Ref-1 in the control of basal p53 stability and activity. Oncogene. 24:3853-63.

100. Bavner A, Matthews J, Sanyal S, Gustafsson JA, Treuter E. (2005) EID3 is a novel EID family member and an inhibitor of CBP-dependent co-activation. Nucleic Acids Res. 33:3561-9.

101. Hirano M, Marti R, Spinazzola A, Nishino I, Nishigaki Y. (2004) Thymidine phosphorylase deficiency causes MNGIE: an autosomal recessive mitochondrial disorder. Nucleosides Nucleotides Nucleic Acids. 23:1217-25.

102. Adham, IM, Sallam MA, Steding G, Korabiowska M, Brinck U et al. (2003) Disruption of the pelota gene causes early embryonic lethality and defects in cell cycle progression. Mol Cell Biol. 23:1470-76.

103. Giebel J, Loster K, Rune GM. (1997) Localization of integrin beta 1, alpha 1, alpha 5 and alpha 9 subunits in the rat testis. Int J Androl. 20:3-9.

104. Chow KL, Hall DH, Emmons SW. (1995) The mab-21 gene of Caenorhabditis elegans encodes a novel protein required for choice of alternate cell fates. Development 121:3615-3626.

105. Castermans D, Wilquet V, Parthoens E, Huysmans C, Steyaert J, Swinnen L, Fryns JP, Van de Ven W, Devriendt K. (2003) The neurobeachin gene is disrupted by a translocation in a patient with idiopathic autism. J Med Genet. 40:352-356.

106. Edgar RC. (2004) MUSCLE: multiple sequence alignment with high accuracy and high throughput. Nucleic Acids Res. 32:1792-1797.

107. Castresana J. (2000) Selection of conserved blocks from multiple alignments for their use in phylogenetic analysis. Mol Biol Evol. 17:540-552.

108. Guindon S, Gascuel O. (2003) A simple, fast, and accurate algorithm to estimate large phylogenies by maximum likelihood. Syst Biol. 52:696-704.

109. Altschul SF, Gish W, Miller W, Myers EW, Lipman DJ. (1990) Basic local alignment search tool. J Mol Biol. 215:403-410.

110. Birney E, Thompson JD, Gibson TJ. (1996) PairWise and SearchWise: finding the optimal alignment in a simultaneous comparison of a protein profile against all DNA translation frames. Nucleic Acids Res. 24:2730-2739.
